# Generalizability and effect measure modification in sibling comparison studies

**DOI:** 10.1007/s10654-022-00844-x

**Published:** 2022-03-21

**Authors:** Arvid Sjölander, Sara Öberg, Thomas Frisell

**Affiliations:** 1 Karolinska Institutet, Nobels väg 12 A, 171 77 Stockholm, Sweden; 2 Karolinska Institutet, Maria Aspmans Gata 30A, 17164 Solna, Sweden

**Keywords:** Bias, Causal inference, Effect measure modification, Sibling comparison study, 92D30, 92C60, 92B15

## Abstract

Sibling comparison studies have the attractive feature of being able to control for unmeasured confounding by factors that are shared within families. However, there is sometimes a concern that these studies may have poor generalizability (external validity) due to the implicit restriction to families that are covariate-discordant, i.e., those families where at least two siblings have different levels of at least one of the covariates (exposure or confounders) under investigation. Even if this selection mechanism has been noted by many authors, previous accounts of the problem tend to be brief. The purpose of this paper is to provide a formal discussion of the implicit restriction to covariate-discordant families in sibling comparison studies. We discuss when and how this restriction may impair the generalizability of the study, and we show that a similar generalizability problem may in fact arise even when all families are covariate-discordant, e.g. even if the exposure is continuous so that all siblings have different exposure levels. We show how this problem can be solved by using a so-called marginal between-within model for estimation of marginal exposure effects. Finally, we illustrate the theoretical conclusions with a simulation study.

## Introduction

Unmeasured confounding is a serious threat to the validity of observational studies. While measured confounders can be accounted for using an array of statistical techniques, the sibling comparison design is capable of also reducing confounding bias from unmeasured and even unknown confounding factors. By comparing differentially exposed siblings within families, rather than between unrelated subjects, the study implicitly controls for all measured and unmeasured factors that siblings from the same family have in common [1, 2]. These factors may, for instance, include early childhood environment and upbringing, and stable parental characteristics such as socioeconomic status and education. A prominent special case is the co-twin control study, which, if restricted to monozygotic twins, completely controls for all heritable genetic factors.

Despite the strong appeal of sibling comparison studies, there is sometimes a concern that these studies may have poor generalizability (external validity) due to various selection mechanisms. One obvious such selection mechanism is the explicit restriction to twins in a co-twin control study; if twins are different from ordinary siblings in important aspects, the exposure effect estimated in twins may not generalize well. Another selection mechanism applies to all sibling comparison studies, through the implicit restriction to families with more than one child. As the aim is to compare siblings within families, only families with more than one child can contribute with information to the study.

A more subtle selection mechanism, which also applies to all sibling comparison studies, is an implicit restriction to families that are covariate-discordant. Here, we use the term ‘covariate’ for both the exposure of interest and for other measured variables (e.g. confounders) that the researcher controls for in the analysis. That a family is ‘covariate-discordant’ means that there are at least two siblings in the family with different levels on at least one of the covariates; this is a necessary condition for the family to be informative about covariate-outcome associations within (conditional on) the family. Conversely, a family is covariate-concordant, and thus non-informative, if all siblings in the family have equal levels on all covariates. When there are no other covariates in the study than the exposure of interest, only exposure-discordant families are informative about the exposure-outcome association within the family. With more covariates in the study the situation is more complex, since a family that is exposure-concordant may be indirectly informative about the within-family exposure-outcome association, to some extent. This happens if the family is discordant on, and thus informative about, at least one of these other covariates, since the within-family associations of all covariates are entangled with each other (technically, they are not likelihood orthogonal). For studies analyzed with conditional logistic regression only families that are ‘doubly discordant’ (at least two siblings in the family with different covariate and outcome levels) are informative [3].

Even if the aforementioned selection mechanisms have been noted by many authors several issues remain unclear, in particular related to the restriction to covariate-discordant families. First, previous accounts of the problem tend to be brief and rarely go beyond stating the obvious (i.e., that selection may reduce generalizability), without commenting on how or to what extent this would influence the particular situation [1, 5–6, 23]. Second, it has been claimed that the problem is moot when the exposure of interest is continuous; for instance, Hutcheon and Harper [7] write that a continuous exposure helps to ‘maintain the generalizability of our findings’. On a superficial level this claim may seem reasonable, since essentially all families with more than one child are exposure/covariate-discordant and thus contribute with information to the study, if the exposure is truly continuous and measured with high accuracy. However, some siblings may still have very similar exposure levels, and are thus close to concordant. One could imagine a gradient in the amount of information provided, where ‘less discordant’ siblings provide less information and the dichotomization into ‘discordant’ and ‘concordant’ are just extreme ends of a continuous spectrum. To our knowledge this has not been investigated and it is unclear if and how such an ‘information gradient’ would affect the generalizability of the study.

The purpose of this paper is to provide a formal discussion of the implicit restriction to covariate-discordant families in sibling comparison studies. We focus on this type of selection mechanism since it is arguably the most subtle and least understood among those mentioned above. The paper is organized as follows: We first establish the notation, definition and assumptions that we will use throughout the paper. We next discuss when and how the restriction to covariate-discordant families may impair the generalizability of the study, and we show that a similar problem may indeed arise even when the exposure is continuous and all families are covariate-discordant. After that we show how this problem can be solved by using a marginal between-within model for estimation of marginal exposure effects, and we illustrate the theoretical results with a simulation study. Finally, we discuss how the presence and magnitude of non-generalizability can be assessed in practice.

## Notation, definitions and assumptions

Let $X_{ij}$ and $Y_{ij}$ be the exposure and outcome of interest, respectively, for sibling *j* in family *i*, for $j=1,\ldots ,n_i$ and $i=1,\ldots ,n$. Let $U_i$ be the full set of confounders, measured or unmeasured, that are shared (i.e., constant) in family *i*. The purpose of sibling comparison studies is to implicitly control for $U_i$ by comparing differentially exposed siblings within the same family. Let $C_{ij}$ be the set of measured non-shared confounders for sibling *j* in family *i*. To keep notation simple we treat $C_{ij}$ as a scalar but in practice it may often be a vector of variables. Finally, let $S_i$ be the indicator of family *i* being selected into (e.g. being informative for) the study; $S_i=1$ for ‘selected’ (informative) and $S_i=0$ for ‘not selected’ (non-informative). For any variable $V_{ij}$ we define the vector ${\mathbf {V}}_i=(V_{i1},\ldots ,V_{in_i})$, the family mean $\overline{V_i}=\sum _{j=1}^{n_i}V_{ij}/n_i$ and the family variance $\overline{\overline{V_i}}=\sum _{j=1}^{n_i}(V_{ij}-\overline{V_i})^2/n_i$.

**Fig. 1:**
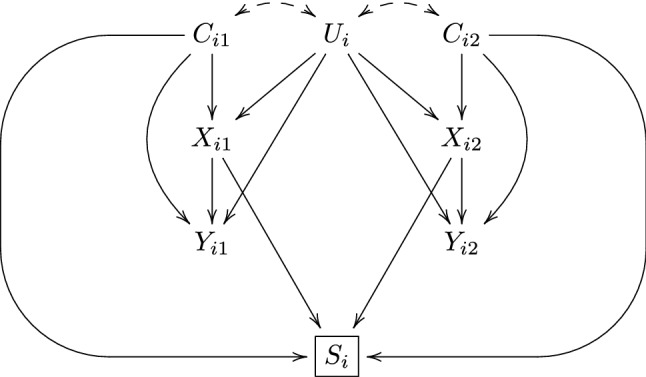
Causal diagram illustrating a sibling comparison study, with restriction to covariate-discordant families

The causal diagram [8] in Fig. 1 illustrates the assumed relations between $U_i$, ${\mathbf {C}}_i$, ${\mathbf {X}}_i$, ${\mathbf {Y}}_i$ and $S_i$, for a family with two siblings, with obvious generalization to larger families. The dashed double-headed arrows between $U_i$ and $(C_{i1},C_{i2})$ indicate that $U_i$ may affect $(C_{i1},C_{i2})$, but also the other way around. The square box around $S_i=1$ indicates the implicit conditioning by selection into the study. The arrows from $(X_{i1},X_{i2})$ and $(C_{i1},C_{i2})$ to $S_i$ represent the restriction to covariate-discordance; a family with size $n_i$ is only selected into (informative for) the study if $(X_{i1},\ldots ,X_{in_i})$ are not all equal or $(C_{i1},\ldots ,C_{in_i})$ are not all equal. As noted in the Introduction we will focus on this restriction and ignore other possible selection mechanisms. For instance, we ignore the possibility that the restriction to families with more than one child may introduce a direct effect of $U_i$ on $S_i$, since the factors that determine whether the parents strive to have more than one child (e.g. socio-economic status) may also confound the exposure and the outcome. We also noted in the Introduction that, in the special case when the outcome is binary and analyzed with conditional logistic regression, only doubly discordant families contribute to the analysis. Hence, in this special case $S_i$ is also directly affected by $Y_{i1},\ldots ,Y_{in_i}$.

The causal diagram in Fig. 1 encodes two important assumptions; no unmeasured non-shared confounding and no carryover effects, i.e., no effect of the exposure and/or outcome of a sibling on the exposure and/or outcome of other siblings. In practice, both assumptions may often be violated to some extent. However, to keep the discussion focused on selection mechanisms and generalizability issues we assume that both assumptions hold and refer to Frisell et al. [9] and Sjölander et al. [10] for further discussions of unmeasured non-shared confounding and carryover effects, respectively.

When analyzing sibling data it is common to assume a fixed effects model on the form

1 $$\begin{aligned} g\{E(Y_{ij}|U_i,X_{ij},C_{ij})\}=\beta _{0i}+\beta _X X_{ij}+\beta _C C_{ij}, \end{aligned}$$

where $E(Y_{ij}|U_i,X_{ij},C_{ij})$ is the conditional mean of the outcome, given $(U_i,X_{ij},C_{ij})$ and *g* is an appropriate link function, typically the identity link, the log link or the logit link. Using the logit link leads to what is often referred to as ‘conditional logistic regression’. The term ‘fixed’ refers to the intercept $\beta _{0i}$, which is a categorical parameter with a fixed level per family. This intercept is intended to absorb, and thereby control for, the shared confounders $U_i$. The parameter $\beta _X$ measures the conditional association between $X_{ij}$ and $Y_{ij}$, given $U_i$ and $C_{ij}$. In the absence of unmeasured non-shared confounders, $\beta _X$ has a causal interpretation as the conditional causal effect of $X_{ij}$ on $Y_{ij}$, given $(U_i,C_{ij})$. This parameter is usually estimated with conditional maximum likelihood; we refer to the Appendix for details.

## Covariate-discordance and effect measure modification by shared confounders

To see intuitively how the restriction to covariate-discordant families may cause generalizability problems, note that the shared confounders are statistically associated with the selection into the study since $U_i$ has an indirect effect on $S_i$ in Fig. 1 mediated through $(X_{i1},\ldots ,X_{in_i})$, and possibly also through $(C_{i1},\ldots ,C_{in_i})$. As a result, those families that are selected into the study will generally have a different distribution of the shared confounders than those not selected into the study. If the shared confounders also modify the effect of the exposure on the outcome, then the observed effect among the covariate-discordant families will not generally be the same as the effect among the covariate-concordant families, and will thus not generalize to the whole population of both covariate-discordant and covariate-concordant families.

To quantify the problem, and to show that a similar problem may arise even for continuous exposures, we consider the fixed effects model (1). For pedagogical purposes we temporarily ignore the measured non-shared confounders $C_{ij}$, so that covariate-discordance/concordance is equivalent with exposure-discordance/concordance, and the assumed model is given by

2 $$\begin{aligned} g\{E(Y_{ij}|U_i,X_{ij})\}=\beta _{0i}+\beta _X X_{ij}. \end{aligned}$$

It is easy to show (see the Appendix) that the fixed effects model estimate $\widehat{\beta _X}$ only draws information from the exposure-discordant families, as expected by intuition. However, it is important to note that this is a feature of the estimation process, not of the model *per se*, which makes no reference to exposure-discordance/concordance. The model conditions on $U_i$, not on exposure-discordance, and the within-family effect $\beta _X$ is assumed to be constant across all levels of $U_i$, i.e., it is assumed that there is no effect measure modification by the shared confounders. Hence, if the model is correct the restriction to exposure-discordant families in the estimation process does not cause any generalizability problems.

To see how generalizability problems may nevertheless arise, suppose that model (2) is not correct, since there is in fact effect measure modification by the shared confounders. Thus, the true model is given by

3 $$\begin{aligned} g\{E(Y_{ij}|U_i,X_{ij})\}=\beta _{0i}+\beta _X(U_i) X_{ij}, \end{aligned}$$

where the parameter $\beta _X$ is a function of $U_i$. Suppose further that *g* is the identity link. It can then be shown (see the Appendix) that, regardless of whether $X_{ij}$ is binary or continuous, the estimate $\widehat{\beta _X}$ under the incorrectly assumed model (2) converges to a weighted average of the $U_i$-specific effects:

4 $$\begin{aligned} \widehat{\beta _X}\rightarrow E\{w(U_i)\beta _X(U_i)\}. \end{aligned}$$

The weights are directly proportional to the conditional variance of $X_{ij}$, given $U_i$:

$$\begin{aligned} w(U_i)=\frac{(n_i-1)\text {Var}(X_{ij}|U_i)}{E\{(n_i-1)\text {Var}(X_{ij}|U_i)\}}. \end{aligned}$$

The expression in (4) shows that all families do not contribute equally to the estimate of $\beta _X$; the families that have levels of $U_i$ associated with a high variability in $X_{ij}$ tend to be more informative. Hence, in the presence of effect measure modification by $U_i$, $\widehat{\beta _X}$ does not converge to the marginal (average) exposure-outcome effect $E\{\beta _X(U_i)\}$ across all families, and does not estimate a parameter that is generally representative of the whole population.

An important special case is when the terms $(n_i-1)\text {Var}(X_{ij}|U_i)$ and $\beta _X(U_i)$ are independent. This happens, for instance, when $n_i$ and $\text {Var}(X_{ij}|U_i)$ are independent of $U_i$, e.g. the shared confounders have no influence on family size or the variability in the exposure. In this special case, it follows from (4) that $\widehat{\beta _X}$ converges to $E\{w(U_i)\}E\{\beta _X(U_i)\}$, which is equal to $E\{\beta _X(U_i)\}$ since $E\{w(U_i)\}=1$. Wooldridge [11] (Section 11.7.3) provided a similar results, but without providing the analytic expression for the large sample limit of $\widehat{\beta _X}$ in (4).

The problem of non-generalizability is most accentuated when some families are exposure-concordant, since the estimate $\widehat{\beta _X}$ gives 0 weight to these families (or, more precisely, it gives 0 weight to the exposure effect $\beta _X(U_i)$ at values of $U_i$ for which $\text {Var}(X_{ij}|U_i)=0$). However, as suggested in the Introduction, this is just an extreme end of a continuous spectrum; a similar problem may arise even if the exposure is continuous and all families are exposure-discordant, as the following example illustrates.

Suppose that $U_i$ is a single variable having a standard (mean 0, standard deviation 1) normal distribution, and that $\beta _X(U_i)=U_i$. For positive values of $U_i$ we have that $\beta _X(U_i)>0$, whereas for negative values of $U_i$ we have that $\beta _X(U_i)<0$. Since $U_i$ is distributed symmetrically around 0 these deviations from 0 cancel out, so that the marginal exposure effect is 0. However, if $n_i$ is independent of $U_i$ we have from (4) that

$$\begin{aligned} \widehat{\beta _X}\rightarrow \frac{E\{\text {Var}(X_{ij}|U_i)U_i\}}{E\{\text {Var}(X_{ij}|U_i)\}}, \end{aligned}$$

which is not generally equal to 0. For instance, suppose that $\text {Var}(X_{ij}|U_i)$ increases from 0.2 to 1.2 as $U_i$ goes from $-\infty$ to $\infty$; specifically, suppose that $\text {Var}(X_{ij}|U_i)=\Phi (U_i)+0.2$, where $\Phi (\cdot )$ is the standard normal CDF. Then, it can be shown numerically that ${\widehat{\beta }}_X$ does not converge to 0, but to 0.4. We return to this example in the ‘Simulation’ section.

The asymptotic limit of ${\widehat{\beta }}_X$ is most straight forward and intuitive for the simple scenario above, where the fixed effects model has an identity link and includes no measured non-shared confounders. In the Appendix we derive the asymptotic limit of ${\widehat{\beta }}_X$ for three other scenarios: the fixed effects model has an identity link and includes one measured non-shared confounder, and the fixed effects model has a log link or a logit link and includes no measured non-shared confounders; for the latter two scenarios we also assume that $X_{ij}$ is binary and $n_i=2$. We show that the asymptotic limit of ${\widehat{\beta }}_X$ is a weighted average of the $U_i$-specific effects for all these scenarios. Although the weights have more complex expressions than for the simple scenario above, a common feature is that the weights increase with $\text {Var}(X_{ij}|U_i)$ (all other factors constant), and are equal to 0 for values of $U_i$ such that $\text {Var}(X_{ij}|U_i)=0$.

## Estimation of marginal effects with marginal between-within models

The generalizability problem discussed in the previous section arises because we use a fixed effects model that assumes no effect measure modification by the shared confounders, when in fact such effect measure modification is present. Although effect measure modification can in principle be modeled by including an interaction (product) term between the exposure and the shared confounders, such interaction is not estimable with conditional maximum likelihood [12]. If the shared confounders were measured, then one could solve the problem by stratifying on the confounders and estimating the conditional effect at each level of these. In practice though, this is typically not possible since the shared confounders are unmeasured to a large extent; indeed, this is the motivation for pursuing a sibling comparison study.

A more feasible way to avoid bias due to effect measure modification is to use a so-called between-within (BW) model. This model comes in two versions; as a marginal [13, 14] or a conditional model [15–17]. Like the fixed effects model, the conditional BW model conditions on the shared confounders, and assumes no effect measure modification by these. In contrast, the marginal BW model marginalizes over the shared confounders, and is thus ignorant about the presence or absence of effect measure modification.

The central idea in (both marginal and conditional) BW models is to assume that the shared confounders $U_i$ can be represented by a measurable ‘proxy variable’ $D_i$, which is a function of the vectors $({\mathbf {X}}_i,{\mathbf {C}}_i)$. Technically, ${\mathbf {X}}_i$ and ${\mathbf {C}}_i$ are assumed to be conditionally independent of $U_i$, given $D_i$:

5 $$\begin{aligned} U_i\bot ({\mathbf {X}}_i,{\mathbf {C}}_i)|D_i; \end{aligned}$$

we refer to assumption (5) as the ‘BW assumption’. The proxy variable $D_i$ is often taken to be the vector of means $(\overline{X_i},{\overline{C}}_i)$; however, to make the BW assumption more realistic we may also include, for instance, the variances $(\overline{\overline{X_i}},\overline{\overline{C_i}})$ [17]. In some studies the family size $n_i$ is likely related to the shared confounders $U_i$. This may for instance be the case in developing countries, where the number of children is often strongly related to familial socio-economic status. In such situations one may include $n_i$ in $D_i$ to make the BW assumption more realistic. In some special cases the BW assumption is guaranteed to hold. In particular, in the absence of measured non-shared confounders the BW assumption holds with $D_i=(\overline{X_i},\overline{\overline{X_i}},n_i)$ when $X_{ij}$ has a normal distribution, and with $D_i=(\overline{X_i},n_i)$ when $X_{ij}$ has a Bernoulli distribution (e.g. when $X_{ij}$ is binary), regardless of how $U_i$ is distributed (see the Appendix).

Under the BW assumption we can estimate the marginal effect of taking the exposure from, say, $x=0$ to $x=1$ in the target population with a marginal BW model, using a five-step procedure. We give a brief explanation of the procedure here, and refer to Sjölander [14] for a more rigorous description. In the first step we fit a marginal BW model on the form

6 $$\begin{aligned} E(Y_{ij}|D_i,X_{ij},C_{ij})=h(D_i,X_{ij},C_{ij};\beta ), \end{aligned}$$

where *h* is a regression function and $\beta$ is a vector of model parameters. A standard linear BW model assumes that $D_i=(\overline{X_i},{\overline{C}}_i)$ and

$$\begin{aligned} E(Y_{ij}|D_i,X_{ij},C_{ij})=\beta _0+\beta _XX_{ij}+\beta _{{\overline{X}}}\overline{X_i}+\beta _CC_{ij}+\beta _{{\overline{C}}}{\overline{C}}_i. \end{aligned}$$

In this model, the coefficients for $\overline{X_i}$ and $X_{ij}$ are referred to as the ‘between-’ and ‘within-effect’ of the exposure, respectively; thus the term ‘BW model’. In the second step we manipulate the observed data by replacing each subject’s factual exposure level with the fixed level 0, but without changing the factual levels of $C_{ij}$ and $D_i$. In the third step we use the fitted model to predict the outcome for each subject in the manipulated data, i.e., for each observed level of $C_{ij}$ and $D_i$. For a given subject, this prediction is an estimate of the counterfactual outcome, had the exposure been set to 0 for that subject while holding the values of $C_{ij}$ and $D_i$ constant. In the fourth step we average these predictions to obtain an estimate of the mean outcome, had the exposure been set to 0 for all subjects. Finally, in the fifth step we repeat the procedure for $x=1$, and contrast the estimated counterfactual means for $x=0$ and $x=1$ to obtain an estimate of the marginal exposure effect. This estimation procedure is a form of regression standardization [18], and can easily be carried out with, for instance, the package stdReg in R [19, 20]. Asymptotic confidence interval and p-values for the estimates can be computed with standard theory for estimating equations as described by Sjölander [14], and can also be obtained from the stdReg package.

We emphasize three important points regarding the model and estimation procedure outlined above. First, although the marginal BW model does not explicitly include (condition on) the shared confounders, it implicitly controls for these through the proxy variable $D_i$. Second, in contrast to the fixed effects model (1), the marginal BW model (6) makes no assumption about the presence or absence of effect measure modification by the shared confounders. Thus, the estimated marginal exposure effect is (asymptotically) unbiased also when such effect measure modification is present, provided that the marginal BW model (6) is correctly specified and the BW assumption (5) holds. Third, standard regression models are often kept simple to make the results transparent and interpretable. However, this is not necessary for the marginal BW model, since the fitting of this model is just the first step in the five-step estimation procedure, and the end product of the procedure (the counterfactual mean outcome) will be no less interpretable if the underlying model is complex than if the model is simple. Hence, to ensure that the model is realistic one may use a relatively complex model specification, including, for instance, splines and interaction terms. We illustrate these points in the next section with a simulation.

## Simulation

In this section we present the results from a simulation study, demonstrating the conclusions and methods from previous sections. We simulated samples of families with 1, 2, 3 or 4 siblings using probabilities 0.2, 0.5, 0.2, and 0.1 to match the distribution of siblings in Sweden [21]. We ignored non-shared confounders $C_{ij}$ and generated the shared confounders, the exposure and the outcome from the model

7 $$\begin{aligned} \left. \begin{array}{rcl} U_i &{} \sim &{} N(0,1)\\ X_{ij}|U_i &{} \sim &{} N\{U_i,\Phi (U_i)+0.2\}\\ g\{E(Y_{ij}|U_i,X_{ij}\}&{}=&{}U_i+\beta _X(U_i)X_{ij} \end{array}\right\} \end{aligned}$$

We generated both a continuous outcome from a normal distribution with unit variance, for which *g* was the identity link, and a binary outcome, for which *g* was the logit link. The parameter $\beta _X(U_i)$ determines the degree of effect measure modification by $U_i$. We considered three scenarios; $\beta _X(U_i)=0$ (no effect measure modification), $\beta _X(U_i)=U_i$ (positive effect measure modification) and $\beta _X(U_i)=-U_i$ (negative effect measure modification). In the first scenario the exposure effect is 0, both conditionally and marginally over $U_i$. In the other two scenarios the conditional exposure effect depends on $U_i$, but the marginal exposure effect is still 0 since positive and negative conditional effects cancel out.

It can be shown (see the Appendix) that under the conditional (on $U_i$) model in (7) the BW assumption (5) holds with $D_i=(\overline{X_i},\overline{\overline{X_i}})$. However, the true regression function $h(D_i,X_{ij};\beta )$ in (6) is rather non-standard and unintuitive. We emphasize that this complexity is a consequence of using a simple conditional model. Alternatively, we could have started by formulating a simple marginal model, which would typically correspond to a complex conditional model. In real scenarios there is no a priori reason why either model would be more plausible than the other.

We generated 1000 samples of $n=1000$ families each from the model in (7). For each sample we estimated the conditional exposure effect $\beta _X$ in the fixed effects model (2), using the gee function in the drgee package in R. This model is correct when there is no effect measure modification ($\beta _X(U_i)=0$) but otherwise incorrect. We also estimated the marginal effect of increasing the exposure from $x=0$ to $x=1$ with two different marginal BW models, fitted with the stdGlm function in the stdReg package. For this marginal effect we used the same link function as in the fixed effects model, i.e. an identity link for the continuous outcome and a logit link for the binary outcome. The first BW model was the standard model

$$\begin{aligned} g\{E(Y_{ij}|D_i,X_{ij})\}=\beta _0+\beta _XX_{ij}+\beta _{{\overline{X}}}\overline{X_i}. \end{aligned}$$

This model incorrectly assumes that the BW assumption (5) holds with $D_i=\overline{X_i}$, and that the relation between $Y_{ij}$ and $(D_i,X_{ij})$ is linear, on the scale defined by the link function *g*. The second BW model was defined as

8 $$\begin{aligned}E(Y_{ij}|D_i,X_{ij})=&\beta _0+\beta _XX_{ij}+\beta _{{\overline{X}}}s(\overline{X_i})+\beta _{\overline{{\overline{X}}}}s(\overline{\overline{X_i}})\nonumber \\\ &+\beta _{X{\overline{X}}}X_{ij}s(\overline{X_i})+\beta _{X\overline{{\overline{X}}}}X_{ij}s(\overline{\overline{X_i}}), \end{aligned}$$

where $s(\overline{X_i})$ and $s(\overline{\overline{X_i}})$ are natural cubic spline functions of $\overline{X_i}$ and $\overline{\overline{X_i}}$, with knots at the three quartiles in their sample distributions. This model correctly assumes that the BW assumption (5) holds with $D_i=(\overline{X_i},\overline{\overline{X_i}})$, but uses spline functions to approximate the correct regression function $h(D_i,X_{ij};\beta )$. For the continuous outcome (i.e. when *g* was the identity link) we additionally fitted the correct marginal BW model, as derived in the Appendix. We did not attempt to fit this model for the binary outcome, since the logit link function makes the model very complex and computationally demanding.

For each fitted model we computed the mean and standard deviation of the estimates over the 1000 samples, together with the mean standard error as obtained from the gee and stdGlm functions, and the empirical coverage probability of the 95% confidence interval estimate±1.96$\times$standard error. R code for the simulation is given in the Appendix.

Table 1 shows the result. In the absence of effect measure modification by $U_i$, all estimates are virtually unbiased (mean estimates equal to 0). However, in the presence of negative and positive effect measure modification by $U_i$, the mean estimates are equal to $-0.4$ and 0.4 for the linear fixed effects model, and $-0.35$ and 0.07 for the logistic fixed effects model, thus biased to various degree. The linear standard BW model has the same degree of bias as the linear fixed effects model for these scenarios, whereas the logistic standard BW model has considerably less bias (mean estimates $-0.07$ and 0.02) than the logistic fixed effects model. The linear spline BW model is virtually unbiased (mean estimates 0.01 and $-0.01$), whereas there appears to be a slight bias in the logistic spline BW model (mean estimates $-0.04$ and $-0.03$). Finally, the correct linear BW model appears to be virtually unbiased (mean estimates 0.01 and $-0.01$). All mean standard errors agree well with the corresponding standard deviations of the estimates, even when the estimates are badly biased. This is expected, given that the gee and stdGlm functions provide robust ‘sandwich’ standard errors, which do not rely on the specified model being correct [22]. However, the 95% confidence intervals only have close to nominal 95% coverage for those scenarios where the estimate is almost unbiased. This is also not surprising, since any asymptotic bias in the estimate, even if minuscule, forces the coverage probability of the confidence interval estimate$\pm 1.96\times$standard error to 0 as the sample size goes to infinity.

Notably, the standard deviation of the estimates from the linear spline BW model are no larger than the standard deviation of the corresponding estimates from the correct linear BW model. This indicates that no statistical efficiency was lost by approximating the correct model with splines. The standard deviation of the estimates from the logistic BW models are considerably smaller than the standard deviation of the corresponding estimates from the logistic fixed effects model. This indicates that, for the logistic link function, the BW model does not only facilitate estimation of marginal effects, but also gives higher statistical efficiency.

In practice, it is unlikely that the analyst would be able to correctly specify a non-standard and unintuitive model such as the true regression function $h(D_i,X_{ij};\beta )$ in our simulation. In contrast, even though the spline model in (8) has a rather complex mathematical expression, it only contains standard spline functions that are available in all major statistical software. It is thus reassuring that the spline BW models gave close to unbiased estimates in our simulation, since it indicates that the analyst may not need to specify the true model correctly; using standard spline functions as an approximation may suffice.

**Table 1:** Simulation results. Mean and standard deviation (sd) of the estimated marginal exposure effect, and mean standard error (se) and coverage probability (cp) of corresponding 95% confidence interval, for the fixed effects model and marginal BW models. The true marginal effect is 0 for all scenarios

	Linear model				Logistic model			
	Mean	sd	se	cp	Mean	sd	se	cp
*No effect measure modification*								
Fixed effects model	0.00	0.04	0.03	0.94	0.00	0.08	0.08	0.95
Standard BW model	0.00	0.04	0.03	0.94	0.00	0.02	0.02	0.95
Spline BW model	0.00	0.04	0.05	0.96	0.00	0.02	0.02	0.95
Correct BW model	0.00	0.04	0.04	0.94	−	−	−	
*Negative effect measure modification*								
Fixed effects model	−0.40	0.06	0.06	0.00	−0.35	0.07	0.08	0.00
Standard BW model	−0.40	0.06	0.06	0.00	−0.07	0.01	0.01	0.00
Spline BW model	0.01	0.06	0.06	0.94	−0.04	0.02	0.02	0.46
Correct BW model	0.01	0.08	0.06	0.93	−	−	−	−
*Positive effect measure modification*								
Fixed effects model	0.40	0.06	0.06	0.00	0.07	0.08	0.08	0.83
Standard BW model	0.40	0.06	0.06	0.00	0.02	0.02	0.02	0.84
Spline BW model	−0.01	0.06	0.06	0.95	−0.03	0.02	0.02	0.71
Correct BW model	−0.01	0.06	0.05	0.94	−	−	−	−

## Assessing the presence and magnitude of non-generalizability

Even though the issue of generalizability in sibling comparison studies has received little formal attention, several authors have proposed informal methods to assess the magnitude of the problem. D’Onofrio et al [23] and Class et al [24] used sibling comparison designs to study the effects of preterm birth and fetal growth restrictions on mortality and psychiatric morbidity. As a sensitivity analysis they estimated the exposure-outcome associations using ordinary (i.e. not fixed effects) regression models, fitted separately to families with two or more children (informative for their sibling comparisons) and to families with only one child (non-informative). They obtained similar estimates in the two groups, which they took as evidence for generalizability of sibling comparison estimates from the former group to the latter. Gebremedhin et al [25] used a sibling comparison design to study the effect of interpregnancy interval on hypertensive disorder. As a sensitivity analysis they compared the distribution of measured covariates (e.g. maternal age at first birth, marital status, ethnicity) between families with three or more children (informative for their sibling comparisons) and families with one or two siblings (non-informative). Like D’Onofrio et al [23] and Class et al [24] they observed no major differences between the groups, which they took as evidence for generalizability.

Although such sensitivity analyses may be informative to some extent, they do not provide definitive evidence. It is possible that both the marginal (over the shared confounders) exposure-outcome association and the distribution of measured covariates are similar between the informative and non-informative families, yet the conditional (on the shared confounders) exposure-association is different, and vice versa. Furthermore, such sensitivity analyses are only useful to the extent that there is a clear-cut between the informative and non-informative families. Families with only one child are clearly non-informative in these studies, as well as families with two children in the study by Gebremedhin et al. However, we have shown that for continuous exposures such as interpregnancy interval there is also a gradient in the information provided, where ‘less discordant’ siblings provide less information. This feature is not addressed in the sensitivity analyses by D’Onofrio et al [23], Class et al [24] and Gebremedhin et al [25] .

An alternative way of assessing the potential for non-generalizability is to consider the underlying mechanisms of the problem. We have shown that non-generalizability arises in sibling comparison studies because of effect measure modification by the shared familial confounders, and we have argued that the problem is compounded if the variability in the exposure depends on the shared confounders. In many studies, some of the shared confounders are measured, which makes it possible to partly assess these mechanisms. As a concrete example, Gebremedhin et al [25] provided data on interpregnancy interval categorized into 7 categories, for Caucasian and non-Caucasian mothers separately; we have reproduced these data in Table 2. A standard $\chi ^2$-test gives a p-value less than $2.2\times 10^{-16}$ for these data; thus, ethnicity is associated with interpregnancy interval. A common measure of variation for nominal variables is the HREL index [26], which is a scaled version of the Shannon entropy. Computing this measure for the data in Table 2 gives very similar figures, 0.88 and 0.90, for the Caucasian and non-Caucasian mothers. This indicates that, although ‘ethnicity’ may be an important shared confounder in the study by Gebremedhin et al [25], it is not plausibly a major source of non-generalizability for their sibling comparisons. If no major differences are found in exposure-variation for any other measured non-shared confounders, then this may taken as evidence for generalizability of the sibling comparison estimates. A similar caveat as above applies here as well though; it is possible that the exposure has similar variation across levels of all measured confounders, but varies strongly across levels of one or several unmeasured confounders.

**Table 2:** Distribution of interpregnancy interval (months) for Caucasian and non-Caucasian mothers, from Gebremedhin et al [25]

	0–5	6–11	12–17	18–23	24–59	60–119	$\ge$120
Caucasian	12299	37050	42262	31413	64944	17801	3304
non-Caucasian	4249	8026	8266	5939	13965	3979	640

Yet another way to assess the magnitude of non-generalizability is to fit both a fixed effects model and a marginal BW model, and compare the estimates. If these are similar, then this may be taken as evidence for generalizability of the fixed effects model estimate. The converse is more questionable though since there could be several explanations for a difference between the estimates, including bias due to misspecification of the marginal BW model, violation of the BW assumption, or (e.g. for logistic models) non-collapsibility of the chosen effect measure [27]. The fit of the marginal BW model can be assessed with standard diagnostic tools, and the model can be refined until a reasonable fit is achieved. If some of the shared confounders are measured, as in the study by Gebremedhin et al [25], one can verify that that BW assumption holds with respect to these, for the particular choice of $D_i$. However, this does not guarantee that the assumption holds with respect to the unmeasured confounders.

In summary, even though the presence and magnitude of non-generalizability can be assessed empirically, such empirical tests can only provide limited evidence. Hence, when judging whether a particular study may suffer from substantial generalizability problems it is also important to use subject matter knowledge about the situation at hand. In some situations one may have a priori reason to believe that there is no substantial effect measure modification by the shared confounders, or that the variation in the exposure is fairly constant across the shared confounders, in which case one may conclude that the sibling comparison estimates are likely to generalize well. However, if such subject matter knowledge is lacking, and the empirical tests discussed above are either not applicable or deemed unreliable, then one should be rather cautious to generalize the results from the sibling comparison study to the whole population.

## Discussion

The sibling comparison design is an important component in the epidemiologic toolbox. However, it has subtle features that are not present in simpler designs, which must be properly understood in order to interpret the results correctly. We have shown how the selection of covariate-discordant families in sibling comparison studies may affect the generalizability of the results, and that a similar generalizability problem may arise even if all families are covariate-discordant (e.g. if the exposure is continuous) if there is effect measure modification by the shared familial confounders. We have demonstrated that the problem can be solved by using a marginal BW model to estimate the marginal exposure effect.

When the exposure effect varies across levels of confounders (or other covariates), stratum-specific effects are often of greater public health interest than the marginal effect, since they can be used to answer more detailed and relevant questions regarding specific subpopulations (e.g. patients with certain characteristics). However, as noted in Section ‘Estimation of marginal effects with marginal between-within models’ it is typically not possible to stratify on all the shared confounders in sibling comparison studies, since these are unmeasured to a large extent. Thus, it seems like the best one could hope for is an approximately unbiased estimate of an effect that is representative for the aggregated population, e.g. a marginal exposure effect.

In our simulation, we used a continuous exposure. Marginal BW models can be used with binary exposures as well since the underlying theory for the model, as described briefly in this paper and more thoroughly by Sjölander [14], makes no assumption about whether the exposure is binary or continuous. However, when the exposure is binary the marginal BW model tends to make strong extrapolation outside the observed data, and may thus be sensitive to the parametric model assumptions. Essentially, this is because when the exposure is binary the value of the proxy variable $D_i$ typically restricts the set of possible exposure values for each individual sibling in the family. For instance, suppose that $D_i$ is taken to be the exposure mean $\overline{X_i}$, and observed to be equal to 1 in a particular family. Then, the individual exposure value $X_{ij}$ must also be equal to 1 for all siblings in that family. Thus, when predicting the counterfactual outcome for an individual *j* in that family, had $X_{ij}$ been hypothetically set to 0, the model has to rely completely on information from other families with $\overline{X_i}\ne 1$. We provide a more detailed discussion of marginal BW models for binary exposures in the Appendix.

## A Conditional maximum likelihood under the fixed effects model (1)

When using conditional maximum likelihood estimation of $\beta _X$ in model (1), the conditional conditional distribution of $Y_{ij}$, given $(U_i,X_{ij})$, is assumed to be on the form

$$\begin{aligned} p(Y_{ij}|U_i,X_{ij})=\text {exp}\left\{ \frac{Y_{ij}\theta _{ij}-A(\theta _{ij})}{\phi }+c(Y_{ij},\phi )\right\} , \end{aligned}$$

where the canonical parameter $\theta _{ij}$ is equal to $\beta _{0i}+\beta _X X_{ij}$. It follows that

$$\begin{aligned} p({\mathbf {Y}}_i|U_i,{\mathbf {X}}_i)= & {} \prod _{j=1}^{n_i}p(Y_{ij}|U_i,X_{ij})\\= & {} \prod _{j=1}^{n_i}\text {exp}\left\{ \frac{Y_{ij}\theta _{ij}-A(\theta _{ij})}{\phi }+c(Y_{ij},\phi )\right\} , \end{aligned}$$

where the first equality follows from the fact that $(X_{i1},Y_{i1},C_{i1}),\ldots ,(X_{in_i},Y_{in_i},C_{in_i})$ are conditionally independent, given $U_i$, under causal diagram in Fig. 1. Inference is conditioned on $n_i{\overline{Y}}_i$, which is a sufficient statistic for $\beta _{0i}$. The conditional maximum likelihood contribution for set *i* becomes equal to

$$\begin{aligned} p({\mathbf {Y}}_i|U_i,{\mathbf {X}}_i,n_i{\overline{Y}}_i)= & {} \frac{p({\mathbf {Y}}_i|U_i,{\mathbf {X}}_i)}{\sum _{{\mathbf {Y}}_i\sim n_i{\overline{Y}}_i}p({\mathbf {Y}}_i|U_i,{\mathbf {X}}_i)}\\= & {} \frac{\text {exp}\{\sum _{j=1}^{n_i}Y_{ij}X_{ij}\beta _X+c(Y_{ij},\phi )\}}{\sum _{{\mathbf {Y}}_i\sim n_i{\overline{Y}}_i}\text {exp}\{\sum _{j=1}^{n_i}Y_{ij}X_{ij}\beta _X+c(Y_{ij},\phi )\}}. \end{aligned}$$

For an exposure-concordant sibling set with $X_{i1}=\ldots X_{in_i}$, the conditional likelihood contribution for set *i* simplifies to

$$\begin{aligned} \frac{\text {exp}\{\sum _{j=1}^{n_i}c(Y_{ij},\phi )\}}{\sum _{{\mathbf {Y}}_i\sim n_i{\overline{Y}}_i}\text {exp}\{\sum _{j=1}^{n_i}c(Y_{ij},\phi )\}}. \end{aligned}$$

This does not depend on $\beta _X$, hence the exposure-concordant sibling sets are non-informative.

## B Asymptotic limit of $\widehat{\beta _X}$ under effect measure modification by $U_i$

If *g* is the identity link, then $Y_{ij}$ is assumed to have a normal distribution with mean $\mu _{ij}=\beta _{0i}+\beta _X X_{ij}$ and constant variance $\sigma ^2$, given $(U_i,X_{ij})$, so that $n_i{\overline{Y}}_i$ has a normal distribution with mean $n_i\overline{\mu _i}$ and variance $n_i\sigma ^2$, given $(U_i,{\mathbf {X}}_i)$. It follows that

$$\begin{aligned}&p({\mathbf {Y}}_i|U_i,{\mathbf {X}}_i,n_i{\overline{Y}}_i)\\&\quad =\frac{p({\mathbf {Y}}_i|U_i,{\mathbf {X}}_i)}{\sum _{{\mathbf {Y}}_i\sim n_i{\overline{Y}}_i}p({\mathbf {Y}}_i|U_i,{\mathbf {X}}_i)}\\&\quad =\frac{\prod _{j=1}^{n_i}(2\pi \sigma ^2)^{-1/2}\text {exp}\{-(Y_{ij}-\mu _{ij})^2/(2\sigma ^2)\}}{(2\pi n_i\sigma ^2)^{-1/2}\text {exp}\{-(n_i{\overline{Y}}_i-n_i\overline{\mu _i})^2/(2n_i\sigma ^2)\}}\\&\quad =\frac{(2\pi \sigma ^2)^{-n_i/2}\text {exp}\{-\sum _{j=1}^{n_i}(Y_{ij}-\mu _{ij})^2/(2\sigma ^2)\}}{(2\pi n_i\sigma ^2)^{-1/2}\text {exp}\{-(n_i{\overline{Y}}_i-n_i\overline{\mu _i})^2/(2n_i\sigma ^2)\}}\\&\quad =\frac{(2\pi \sigma ^2)^{-n_i/2}}{(2\pi n_i\sigma ^2)^{-1/2}}\text {exp}\\&\qquad \left[ \frac{-\sum _{j=1}^{n_i}\{(Y_{ij}-{\overline{Y}}_i)-(\mu _{ij}-\overline{\mu _i})\}^2}{2\sigma ^2}\right] \\&\quad =\frac{(2\pi \sigma ^2)^{-n_i/2}}{(2\pi n_i\sigma ^2)^{-1/2}}\text {exp}\\&\qquad \left[ \frac{-\sum _{j=1}^{n_i}\{(Y_{ij}-{\overline{Y}}_i)-\beta _X(X_{ij}-\overline{X_i})\}^2}{2\sigma ^2}\right] . \end{aligned}$$

Hence, the conditional maximum likelihood score contribution from set *i* is

9 $$\begin{aligned} S_i(\beta _X)=\sum _{j=1}^{n_i}(X_{ij}-\overline{X_i})\{(Y_{ij}-{\overline{Y}}_i)-\beta _X (X_{ij}-\overline{X_i})\}. \end{aligned}$$

If *g* is the log link, then $Y_{ij}$ is assumed to have a Poisson distribution with mean $\mu _{ij}=\text {exp}(\beta _{0i}+\beta _X X_{ij})$, given $(U_i,X_{ij})$, so that $n_i{\overline{Y}}_i$ has a Poisson distribution with mean $n_i\overline{\mu _i}$, given $(U_i,{\mathbf {X}}_i)$. It follows that

$$\begin{aligned}&p({\mathbf {Y}}_i|U_i,{\mathbf {X}}_i,n_i{\overline{Y}}_i)\\&\quad =\frac{p({\mathbf {Y}}_i|U_i,{\mathbf {X}}_i)}{\sum _{{\mathbf {Y}}_i\sim n_i{\overline{Y}}_i}p({\mathbf {Y}}_i|U_i,{\mathbf {X}}_i)}\\&\quad =\frac{\prod _{j=1}^{n_i}(Y_{ij}!)^{-1}\mu _{ij}^{Y_{ij}}\text {exp}(-\mu _{ij})}{(n_i{\overline{Y}}_i!)^{-1}(n_i\overline{\mu _i})^{n_i{\overline{Y}}_i}\text {exp}(-n_i\overline{\mu _i})}\\&\quad =\frac{\prod _{j=1}^{n_i}(Y_{ij}!)^{-1}}{(n_i{\overline{Y}}_i!)^{-1}}\prod _{j=1}^{n_i}\left( \frac{\mu _{ij}}{n_i\overline{\mu _i}}\right) ^{Y_{ij}}\\&\quad =\frac{\prod _{j=1}^{n_i}(Y_{ij}!)^{-1}}{(n_i{\overline{Y}}_i!)^{-1}}\prod _{j=1}^{n_i}\left( \frac{\text {exp}(\beta _X X_{ij})}{\sum _{j=1}^{n_i}\text {exp}(\beta _X X_{ij})}\right) ^{Y_{ij}}. \end{aligned}$$

Hence, the conditional maximum likelihood score contribution from set *i* is

$$\begin{aligned} S_i(\beta _X)=\sum _{j=1}^{n_i}Y_{ij}\left\{ X_{ij}-\frac{\sum _{j=1}^{n_i}X_{ij}\text {exp}(\beta _X X_{ij})}{\sum _{j=1}^{n_i}\text {exp}(\beta _X X_{ij})}\right\} . \end{aligned}$$

When $n_i=2$ and $X_{ij}$ is binary, this simplifies to

10 $$\begin{aligned} S_i(\beta _X)= & {} \sum _{j=1}^{2}Y_{ij}\left\{ X_{ij}\frac{1}{1+\text {exp}(\beta _X)}\right. \nonumber \\&\left. +(1-X_{ij})\frac{\text {exp}(\beta _X)}{1+\text {exp}(\beta _X)}\right\} I(X_{i1}\ne X_{i2}). \end{aligned}$$

If *g* is the logit link, then $Y_{ij}$ is assumed to have a Bernoulli distribution with mean $\mu _{ij}=\text {expit}(\beta _{0i}+\beta _X X_{ij})$, given $(U_i,X_{ij})$, so that

$$\begin{aligned}&p({\mathbf {Y}}_i|U_i,{\mathbf {X}}_i,n_i{\overline{Y}}_i)\\&\quad =\frac{p({\mathbf {Y}}_i|U_i,{\mathbf {X}}_i)}{\sum _{{\mathbf {Y}}_i\sim n_i{\overline{Y}}_i}p({\mathbf {Y}}_i|U_i,{\mathbf {X}}_i)}\\&\quad =\frac{\prod _{j=1}^{n_i}\mu _{ij}^{Y_{ij}}(1-\mu _{ij})^{1-Y_{ij}}}{\sum _{{\mathbf {Y}}_i\sim n_i{\overline{Y}}_i}\prod _{j=1}^{n_i}\mu _{ij}^{Y_{ij}}(1-\mu _{ij})^{1-Y_{ij}}}\\&\quad =\frac{\prod _{j=1}^{n_i}\{\mu _{ij}/(1-\mu _{ij})\}^{Y_{ij}}(1-\mu _{ij})}{\sum _{{\mathbf {Y}}_i\sim n_i{\overline{Y}}_i}\prod _{j=1}^{n_i}\{\mu _{ij}/(1-\mu _{ij})\}^{Y_{ij}}(1-\mu _{ij})}\\&\quad =\frac{\prod _{j=1}^{n_i}\text {exp}(\beta _X X_{ij}Y_{ij})}{\sum _{{\mathbf {Y}}_i\sim n_i{\overline{Y}}_i}\prod _{j=1}^{n_i}\text {exp}(\beta _X X_{ij}Y_{ij})}\\&\quad =\frac{\text {exp}(\beta _X \sum _{j=1}^{n_i}X_{ij}Y_{ij})}{\sum _{{\mathbf {Y}}_i\sim n_i{\overline{Y}}_i}\text {exp}(\beta _X \sum _{j=1}^{n_i}X_{ij}Y_{ij})}, \end{aligned}$$

which is the usual conditional logistic regression likelihood. Hence, the conditional maximum likelihood score contribution from set *i* is

$$\begin{aligned} S_i(\beta _X)= & {} \sum _{j=1}^{n_i}X_{ij}Y_{ij}\\&-\frac{\sum _{{\mathbf {Y}}_i\sim n_i{\overline{Y}}_i}\sum _{j=1}^{n_i}X_{ij}Y_{ij}\text {exp}(\beta _X \sum _{j=1}^{n_i}X_{ij}Y_{ij})}{\sum _{{\mathbf {Y}}_i\sim n_i{\overline{Y}}_i}\text {exp}(\beta _X \sum _{j=1}^{n_i}X_{ij}Y_{ij})}. \end{aligned}$$

When $n_i=2$ and $X_{ij}$ is binary, this simplifies to

11 $$\begin{aligned} S_i(\beta _X)=\left\{ \frac{I(Y_{i1}=X_{i1},Y_{i2}=X_{i2})}{1+\text {exp}(\beta _X)}-\frac{I(Y_{i1}=X_{i2},Y_{i2}=X_{i1})\text {exp}(\beta _X)}{1+\text {exp}(\beta _X)}\right\} I(X_{i1}\ne X_{i2}).\nonumber \\ \end{aligned}$$

It follows from standard theory for estimating equations that $\widehat{\beta _X}$ converges to the solution to the equation

$$\begin{aligned} E\{S_i(\beta _X)\}=0. \end{aligned}$$

For the score contribution in (9) we have, under model (3), that

$$\begin{aligned} E\{S_i(\beta _X)|U_i,{\mathbf {X}}_i\}=\{\beta _X (U_i)-\beta _X\}(X_{ij}-\overline{X_i})^2, \end{aligned}$$

so that

$$\begin{aligned} E\{S_i(\beta _X)|U_i\}=\{\beta _X (U_i)-\beta _X\}(n_i-1)\text {Var}(X_{ij}|U_i). \end{aligned}$$

It follows that $\widehat{\beta _X}$ converges to the expression in (4). For the score contribution in (10) we have, under model (3), that

$$\begin{aligned} E\{S_i(\beta _X)|U_i,{\mathbf {X}}_i\}= & {} \frac{E(Y_{ij}|U_i,X_{ij}=0)}{1+\text {exp}(\beta _X)}[\text {exp}\{\beta _X (U_i)\}\\&-\text {exp}(\beta _X)]I(X_{i1}\ne X_{i2}), \end{aligned}$$

so that

$$\begin{aligned} E\{S_i(\beta _X)|U_i\}= & {} \frac{E(Y_{ij}|U_i,X_{ij}=0)}{1+\text {exp}(\beta 
_X)}\\&[\text {exp}\{\beta _X (U_i)\}-\text {exp}(\beta _X)]\text {Var}(X_{ij}|U_i). \end{aligned}$$

It follows that

$$\begin{aligned} \text {exp}(\widehat{\beta _X})\rightarrow E[w(U_i)\text {exp}\{\beta _X(U_i)\}]. \end{aligned}$$

where

$$\begin{aligned} w(U_i)=\frac{E(Y_{ij}|U_i,X_{ij}=0)\text {Var}(X_{ij}|U_i)}{E\{E(Y_{ij}|U_i,X_{ij}=0)\text {Var}(X_{ij}|U_i)\}}. \end{aligned}$$

We have that $w(U_i)$ increases with $\text {Var}(X_{ij}|U_i)$, and that $\text {Var}(X_{ij}|U_i)=0\Rightarrow w(U_i)=0$.

For the score contribution in (11) we have, under model (3), that

$$\begin{aligned}&E\{S_i(\beta _X)|U_i,{\mathbf {X}}_i\}\\&\quad =\frac{p(Y_{ij}=1|U_i,X_{ij}=0)p(Y_{ij}=0|U_i,X_{ij}=1)}{1+\text {exp}(\beta _X)}\\&\qquad [\text {exp}\{\beta _X (U_i)\}-\text {exp}(\beta _X)]I(X_{i1}\ne X_{i2}) \end{aligned}$$

so that

$$\begin{aligned}&E\{S_i(\beta _X)|U_i\}\\&\quad =\frac{p(Y_{ij}=1|U_i,X_{ij}=0)p(Y_{ij}=0|U_i,X_{ij}=1)}{1+\text {exp}(\beta _X)}\\&\qquad [\text {exp}\{\beta _X (U_i)\}-\text {exp}(\beta _X)]\text {Var}(X_{ij}|U_i). \end{aligned}$$

It follows that

$$\begin{aligned} \text {exp}(\widehat{\beta _X})\rightarrow E[w(U_i)\text {exp}\{\beta _X(U_i)\}]. \end{aligned}$$

where

$$\begin{aligned} w(U_i)=\frac{\text {Var}(X_{ij}|U_i)p(Y_{ij}=1|U_i,X_{ij}=0)p(Y_{ij}=0|U_i,X_{ij}=1)}{E\{\text {Var}(X_{ij}|U_i)p(Y_{ij}=1|U_i,X_{ij}=0)p(Y_{ij}=0|U_i,X_{ij}=1)\}}. \end{aligned}$$

Again, we have that $w(U_i)$ increases with $\text {Var}(X_{ij}|U_i)$, and that $\text {Var}(X_{ij}|U_i)=0\Rightarrow w(U_i)=0$.

Consider now the situation where $Y_{ij}$ is assumed to have a normal distribution with mean $\mu _{ij}=\beta _{0i}+\beta _X X_{ij}+\beta _C C_{ij}$ and constant variance $\sigma ^2$, given $(U_i,X_{ij},C_{ij})$, for a scalar covariate $C_{ij}$. Arguing as above, the conditional maximum likelihood score contribution from set *i* is

$$\begin{aligned} S_i(\beta _X,\beta _C)= & {} \sum _{j=1}^{n_i}\left\{ \begin{array}{c}(X_{ij}-\overline{X_i})\\ (C_{ij}-\overline{C_i})\end{array}\right\} \{(Y_{ij}-{\overline{Y}}_i)-\beta _X (X_{ij}-\overline{X_i})\\&-\beta _C (C_{ij}-\overline{C_i})\}. \end{aligned}$$

Suppose now that the true mean of $Y_{ij}$ is $\mu _{ij}=\beta _{0i}+\beta _X(U_i) X_{ij}+\beta ^*_C C_{ij}$. It follows that

$$\begin{aligned}&E\{S_i(\beta _X,\beta _C)|U_i,{\mathbf {X}}_i,{\mathbf {C}}_i\}\\&\quad =\sum _{j=1}^{n_i}\left\{ \begin{array}{c}(X_{ij}-\overline{X_i})\\ (C_{ij}-\overline{C_i})\end{array}\right\} \\&\qquad [\{\beta _X(U_i)-\beta _X\} (X_{ij}-\overline{X_i})-(\beta ^*_C-\beta _C) (C_{ij}-\overline{C_i})] \end{aligned}$$

so that

$$\begin{aligned}&E\{S_i(\beta _X,\beta _C)|U_i\}=(n_i-1)\\&\quad \left[ \begin{array}{c}\{\beta _X(U_i)-\beta _X\}\text {Var}(X_{ij}|U_i)+(\beta ^*_C-\beta _C)\text {Cov}(X_{ij},C_{ij}|U_i) \\ \{\beta _X(U_i)-\beta _X\}\text {Cov}(X_{ij},C_{ij}|U_i)+(\beta ^*_C-\beta _C)\text {Var}(C_{ij}|U_i) \end{array}\right] . \end{aligned}$$

It follows, after some algebra, that

12 $$\begin{aligned} \widehat{\beta _X}\rightarrow E\{w(U_i)\beta _X(U_i)\}. \end{aligned}$$

where

$$\begin{aligned} w(U_i)=\frac{(n_i-1)[\text {Var}(X_{ij}|U_i)E\{\text {Var}(C_{ij}|U_i)\}-\text {Cov}(X_{ij},C_{ij}|U_i)E\{\text {Cov}(X_{ij},C_{ij}|U_i)\}]}{E((n_i-1)[\text {Var}(X_{ij}|U_i)E\{\text {Var}(C_{ij}|U_i)\}-\text {Cov}(X_{ij},C_{ij}|U_i)E\{\text {Cov}(X_{ij},C_{ij}|U_i)\}])}. \end{aligned}$$

As before we have that we have that $w(U_i)$ increases with $\text {Var}(X_{ij}|U_i)$. Furthermore, it follows from the Cauchy-Schwarz inequality that $\text {Cov}(X_{ij},C_{ij}|U_i)\le \sqrt{\text {Var}(X_{ij}|U_i)\text {Var}(C_{ij}|U_i)}$, so that $\text {Var}(X_{ij}|U_i)=0\Rightarrow \text {Cov}(X_{ij},C_{ij}|U_i)=0\Rightarrow w(U_i)=0$.

## C Special cases when the BW assumption is guaranteed to hold

In the absence of measured non-shared confounders we have that

$$\begin{aligned} p(U_i|{\mathbf {X}}_i)= & {} \frac{p({\mathbf {X}}_i|U_i)p(U_i)}{\int p({\mathbf {X}}_i|U_i=u)p(U_i=u)du}\\= & {} \frac{\prod _{j=1}^{n_i}p(X_{ij}|U_i)p(U_i)}{\int \prod _{j=1}^{n_i}p(X_{ij}|U_i=u)p(U_i=u)du}. \end{aligned}$$

Thus, $p(U_i|{\mathbf {X}}_i)$ only depends on ${\mathbf {X}}_i$ through the term $\prod _{j=1}^{n_i}p(X_{ij}|U_i)$. If $X_{ij}|U_i\sim N\{\mu (U_i),\sigma ^2(U_i)\}$ we have that

$$\begin{aligned}&\prod _{j=1}^{n_i}p(X_{ij}|U_i)\\&\quad =\prod _{j=1}^{n_i} \frac{1}{\sqrt{2\pi \sigma ^2(U_i)}}\text {exp}\left[ -\frac{\{X_{ij}-\mu (U_i)\}^2}{2\sigma ^2(U_i)}\right] \\&\quad = \frac{1}{[2\pi \sigma ^2(U_i)]^{n_i/2}}\text {exp}\left( \frac{-n_i\left[ \overline{\overline{X_i}}+ \{\overline{X_i}-\mu (U_i)\}^2\right] }{2\sigma ^2(U_i)}\right) . \end{aligned}$$

If $X_{ij}|U_i\sim \text {Ber}\{p(U_i)\}$ we have that

$$\begin{aligned} \prod _{j=1}^{n_i}p(X_{ij}|U_i)= & {} \prod _{j=1}^{n_i} \{p(U_i)\}^{X_{ij}}\{1-p(U_i)\}^{1-X_{ij}}\\= & {} \{p(U_i)\}^{n_i\overline{X_i}}\{1-p(U_i)\}^{n_i(1-\overline{X_i})}. \end{aligned}$$

## D Correct marginal BW model implied by the conditional model (7)

Under model (7) we have that

$$\begin{aligned} E(Y_{ij}|{\mathbf {X}}_i)= & {} E\{\beta _{0i}+\beta _{X}(U_i)X_{ij}|{\mathbf {X}}_i\}\\= & {} \left\{ \begin{array}{lll}E(U_i|{\mathbf {X}}_i)&{} \text {if}&{} \beta _{X}(U_i)=0\\ E(U_i|{\mathbf {X}}_i)(1+X_{ij})&{} \text {if}&{} \beta _{X}(U_i)=U_i\end{array}\right. \end{aligned}$$

where

$$\begin{aligned}&E(U_i|{\mathbf {X}}_i)\\&\quad =\int u p(U_i=u|{\mathbf {X}}_i)du\\&\quad =\frac{\int up({\mathbf {X}}_i|U_i=u)p(U_i=u)du}{\int p({\mathbf {X}}_i|U_i=u)p(U_i=u)du}\\&\quad =\frac{\int u\prod _{j=1}^{n_i}p(X_{ij}|U_i=u)p(U_i=u)du}{\int \prod _{j=1}^{n_i}p(X_{ij}|U_i=u)p(U_i=u)du}\\&\quad =\frac{\int uq(u,D_i)du}{\int q(u,D_i)du}, \end{aligned}$$

with $$D_i=(\overline{X_i},\overline{\overline{X_i}})$$,

$$\begin{aligned} q(u,D_i)= & {} \frac{1}{[2\pi \{\Phi (u)+0.2\}]^{n_i/2}}\\&\text {exp}\left[ \frac{-n_i\left\{ \overline{\overline{X_i}}+(\overline{X_i}-u)^2\right\} }{2\{\Phi (u)+0.2\}}\right] \phi (u;0,1) \end{aligned}$$

and $\phi (u;\mu ,\sigma ^2)$ being the normal density function with mean $\mu$ and variance $\sigma ^2$. Since $U_i$ only depends on $\mathbf{X }_i$ through $D_i$, it follows that $D_i$ satisfies the BW assumption (5); see Sjölander [14], equation 2.2.

## E Marginal BW models for binary exposures

In this section we provide further discussion of the marginal BW model with a binary exposure. As a motivating example, suppose that $n_i=2$ for all *i*, $X_{ij}\in \{0,1\}$, $C_{ij}=\emptyset$ and $D_i={\overline{X}}_i$. Define $\mu _{x,{\overline{X}}_i}=E(Y_{ij}|X_{ij}=x,{\overline{X}}_i)$ and ${\widetilde{\mu }}_{x,{\overline{X}}_i}=E\{Y_{ij}(x)|{\overline{X}}_i\}$, where $Y_{ij}(x)$ is the potential outcome [8] for sibling *j* in family *i*, if the exposure were set to level *x*. The observed data distribution defines the four parameters $(\mu _{0,0},\mu _{0,0.5},\mu _{1,0.5},\mu _{1,1})$. However, the two parameters $(\mu _{0,1},\mu _{1,0})$ are not well defined since ${\overline{X}}_i=1$ is logically incompatible with $X_{ij}=0$, and ${\overline{X}}_i=0$ is logically incompatible with $X_{ij}=1$. This is analogous to violations of the positivity assumption in the context of inverse probability weighting [28].

For this example, the BW assumption (5) trivially holds, since ${\mathbf {X}}_i$ is a constant (up to the arbitrary ordering of $X_{i1}$ and $X_{i2}$) conditional on $D_i={\overline{X}}_i$, thus conditionally independent of $U_i$, given $D_i$. We further have that ${\widetilde{\mu }}_{x,{\overline{X}}_i}$ and $\mu _{x,{\overline{X}}_i}$ are related through

13 $$\begin{aligned} \left. \begin{array}{lll} {\widetilde{\mu }}_{0,0} = & \mu _{0,0}\\ {\widetilde{\mu }}_{0,0.5} = & \mu _{0,0.5}\\ {\widetilde{\mu }}_{1,0.5} = & \mu _{1,0.5}\\ {\widetilde{\mu }}_{1,1} = &\mu _{1,1} \end{array}\right\} \end{aligned}$$

It follows from results by Sjölander [14] (Supplementary material, Section 3) that, in order for regression standardization to give consistent estimates of marginal causal effects, the regression function $h({\overline{X}}_i,X_{ij};\beta )$ in (6) must be a correct model for ${\widetilde{\mu }}_{x,{\overline{X}}_i}$ when the observed exposure level $X_{ij}$ is replaced in the regression function by the fixed constant *x*. Notably, this requirement must be met **even when the observed** ${\overline{X}}_i$ **is logically incompatible with an observed value** $X_{ij}=x$. This makes clear that regression standardization makes a strong extrapolation when estimating $({\widetilde{\mu }}_{0,1},{\widetilde{\mu }}_{1,0})$, since these are not determined by the observed data distribution. For instance, suppose that we consider two different parametric models $h({\overline{X}}_i,X_{ij};\beta )$, say $M_1$ and $M_2$. Suppose that neither $M_1$ nor $M_2$ imply any restrictions on $(\mu _{0,0},\mu _{0,0.5},\mu _{1,0.5},\mu _{1,1})$. Then, both $M_1$ and $M_2$ fit the observed data equally well, so that the observed data cannot be used to distinguish between the models. Furthermore, the (ML) estimates of $(\mu _{0,0},\mu _{0,0.5},\mu _{1,0.5},\mu _{1,1})$ are equal to the corresponding sample means for both $M_1$ and $M_2$, so that $M_1$ and $M_2$ give the same estimates of $({\widetilde{\mu }}_{0,0},{\widetilde{\mu }}_{0,0.5},{\widetilde{\mu }}_{1,0.5},{\widetilde{\mu }}_{1,1})$ through the relations in (13). However, $M_1$ and $M_2$ may still give different estimates of $({\widetilde{\mu }}_{0,1},{\widetilde{\mu }}_{1,0})$, when $h({\overline{X}}_i,x;\beta )$ is interpreted as a model for ${\widetilde{\mu }}_{x,{\overline{X}}_i}$.

As a concrete example, suppose that we wish to estimate the marginal mean difference

14 $$\begin{aligned} \psi= & E\{Y_{ij}(1)\}-E\{Y_{ij}(0)\}\nonumber \\= & {} \sum _{{\overline{X}}_i\in (0,0.5,1)}({\widetilde{\mu }}_{1,{\overline{X}}_i}-{\widetilde{\mu }}_{0,{\overline{X}}_i})p({\overline{X}}_i), \end{aligned}$$

and consider the following two models:

15 $$\begin{aligned} M_1:&h({\overline{X}}_i,X_{ij};\beta )=\beta _0+\beta _1X_{ij}\nonumber \\&\quad +\beta _2I({\overline{X}}_i=0.5)+\beta _3I({\overline{X}}_i=1) \end{aligned}$$

and

16 $$\begin{aligned} M_2: h({\overline{X}}_i,X_{ij};\beta )=\beta _0+\beta _XX_{ij}+\beta _{{\overline{X}}_i}{\overline{X}}_i+\beta _{X{\overline{X}}}x{\overline{X}}_i. \end{aligned}$$

It is easy to see that neither of these models imply any restrictions on $(\mu _{0,0},\mu _{0,0.5},\mu _{1,0.5},\mu _{1,1})$. However, by interpreting $h({\overline{X}}_i,x;\beta )$ as a model for ${\widetilde{\mu }}_{x,{\overline{X}}_i}$ we have, for $M_1$, that

$$\begin{aligned} {\widetilde{\mu }}_{0,1}= & {} \beta _0+\beta _3=\mu _{1,1}-\mu _{1,0.5}+\mu _{0,0.5}\\ {\widetilde{\mu }}_{1,0}= & {} \beta _0+\beta _1=\mu _{0,0}+\mu _{1,0.5}-\mu _{0,0.5} \end{aligned}$$

and

$$\begin{aligned} \psi =\mu _{1,0.5}-\mu _{0,0.5}, \end{aligned}$$

which is the mean difference among the exposure-discordant pairs. In contrast, for $M_2$ we have that

$$\begin{aligned} {\widetilde{\mu }}_{0,1}= & {} \beta _0+\beta _{{\overline{X}}}=2\mu _{0,0.5}-\mu _{0,0}\\ {\widetilde{\mu }}_{1,0}= & {} \beta _0+\beta _X=2\mu _{1,0.5}-\mu _{1,1}. \end{aligned}$$

and

$$\begin{aligned} \psi= & {} (\mu _{1,0.5}-\mu _{0,0.5})+\{p({\overline{X}}_i=1)-p({\overline{X}}_i=0)\}\\&(\mu _{1,1}-\mu _{1,0.5}-\mu _{0,0.5}+\mu _{0,0}). \end{aligned}$$

Thus, the obtained estimate of $\psi$ depends heavily on whether we use model $M_1$ or model $M_2$.
